# Design of a New Ultracompact Resonant Plasmonic Multi-Analyte Label-Free Biosensing Platform

**DOI:** 10.3390/s17081810

**Published:** 2017-08-06

**Authors:** Francesco Dell’Olio, Donato Conteduca, Maripina De Palo, Caterina Ciminelli

**Affiliations:** Optoelectronic Laboratory, Politecnico di Bari, Via Orabona 4, 70125 Bari, Italy; francesco.dellolio@poliba.it (F.D.); donato.conteduca@poliba.it (D.C.); maripinadepalo@gmail.com (M.D.P.)

**Keywords:** biosensor, plasmonics, label-fee, Bragg grating

## Abstract

In this paper, we report on the design of a bio-multisensing platform for the selective label-free detection of protein biomarkers, carried out through a 3D numerical algorithm. The platform includes a number of biosensors, each of them is based on a plasmonic nanocavity, consisting of a periodic metal structure to be deposited on a silicon oxide substrate. Light is strongly confined in a region with extremely small size (=1.57 μm^2^), to enhance the light-matter interaction. A surface sensitivity *S_s_* = 1.8 nm/nm has been calculated together with a detection limit of 128 pg/mm^2^. Such performance, together with the extremely small footprint, allow the integration of several devices on a single chip to realize extremely compact lab-on-chip microsystems. In addition, each sensing element of the platform has a good chemical stability that is guaranteed by the selection of gold for its fabrication.

## 1. Introduction

Early diagnosis and monitoring of several diseases, such as cancer or cardiovascular diseases, by biological fluids analysis require simultaneous detection of traces of a set of properly-selected protein biomarkers [1]. Since the development of lab-on-chip microsystems implementing this functionality requires chip-scale multi-analyte biosensing platforms integrating a number of biosensors, the research effort on micron/nanoscale biosensor technologies suitable for compact multi-analyte platforms is quickly growing [2].

Label-free optically-based biosensors have demonstrated several advantages when compared to the competing technologies [3]. In particular, the main advantages of these devices are related to strong compactness, high accuracy, and sensitivity, together with low detection limit and wide dynamic range. In optical resonant biosensors changes in the biomarker concentrations can be detected by changes in the optical properties of a resonant cavity with a real-time response [4,5,6,7,8,9]. Due to their very good performance and compactness, optical biosensors can be used for multiplexed analysis of several biomarkers, so enabling the integration of several devices on a single chip, each of them are able to detect a specific biomarker.

Lab-on-chip microsystems [10,11] based on several optical label-free biosensors have been commercialized already, providing a resolution much lower than 100 ng/mL and enabling detection of DNA and RNA strands, proteins, and viruses [12].

Among optical biosensors, a strong research interest in plasmonic configurations, as largely used in the field of ultra-sensitive detection of bio-molecules, e.g., protein biomarkers, has been observed because they allow values of resolution not achievable by the competing technologies [13]. In fact, surface plasmon resonance (SPR) biosensors [14], which have been on the market for more than twenty years, are able to detect a wide range of bio-molecules with a typical resolution less than 1 ng/mL, which is suitable for almost all emerging applications in medicine. Those sensors are based on a properly-functionalized thin gold film where a plasmon resonance, very sensitive to any molecular interaction in close proximity of the functionalization layer, is excited by a light beam passing through a glass prism, according to the well-known Kretschmann configuration [15]. By monitoring the plasmon resonance shift due to the interaction between the functionalization layer and the analyte, the bio-molecules can be detected with high resolution.

However, because SPR instruments are bulky and their miniaturization is challenging due to the optics required to couple light into the gold film, new plasmonics technologies at the nanoscale for biosensing, e.g., those based on localized SPRs excited in metal nanoparticles or plasmonic nanopores and nanoholes in thin metal films, are currently under investigation for lab-on-chip microsystems [16,17,18,19,20,21]. Among those technologies, planar resonant structures where light is confined in surface plasmon polariton (SPP) waveguides [22,23,24] are very advantageous because a planar resonant biosensor can be easily arrayed due to a footprint of a few μm^2^, and exhibits a resolution that is compliant to the requirements of many applications in the field of medicine. In addition, a biosensor in which the light is confined in SPP waveguides exhibits the typical advantages of the integrated optical devices, including the possibility to be manufactured by the standard fabrication tools and techniques widely utilized in micro and nanoelectronics.

SPP waveguides can exhibit either an insulator-metal-insulator (IMI) or a metal-insulator-metal (MIM) configuration [25]. The latter waveguiding structure is the most efficient for subwavelength confinement of light and the most suitable for the manufacturing of the above-mentioned resonant structures [26,27].

Here, we report on the design of a new MIM ultracompact integrated platform, which includes multiple planar label-free plasmonic biosensors for a multiplexed analysis of protein biomarkers.

To our knowledge, the designed multi-analyte biosensing platform is the first one based on SPP planar waveguides.

We have designed the platform to sense three protein biomarkers, but the utilized design technique can be followed and generalized for a larger number of biomarkers. The platform could be used for detection/monitoring of a number of diseases. By way of example, we mention a specific application, i.e., early diagnosis and monitoring of coronary artery diseases by sensing three protein biomarkers, C-reactive protein, β_2_-microglobulin, and adiponectin.

## 2. Platform Configuration and Design

**Figure 1:**
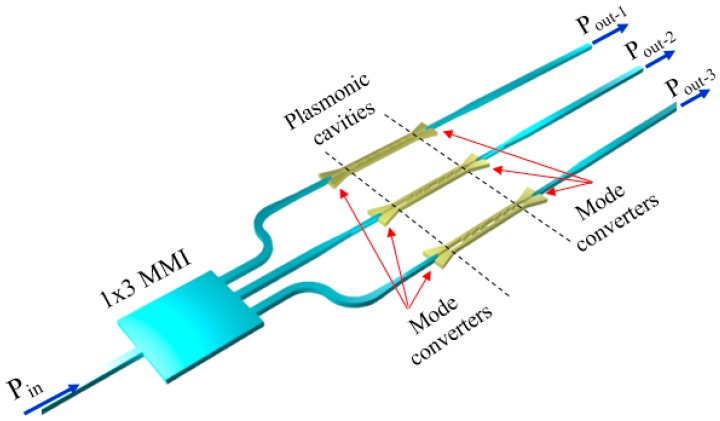
Biosensing platform for the detection and monitoring of several protein biomarkers. Optical fibers that should be coupled to the input and output waveguides are not shown. Figure not to scale.

The configuration of the biosensing platform for detecting and monitoring a number of biomarkers is shown in Figure 1.

The system includes an array of nanoplasmonic biosensors with extremely small size to be functionalized with different receptors in order to detect and monitor specific biomarkers. A mode converter is placed at both the input and output of each biosensor to convert the plasmonic mode confined in the plasmonic cavity into a guided mode propagating in a dielectric waveguide in silicon-on-insulator (SOI) technology, and vice versa. The guiding structure, which can be coupled to a single-mode optical fiber with high efficiency (> 70% [28]), is a low-loss (~ 0.5 dB/cm [29]) standard Si wire having a width of 500 nm and a height of 220 nm.

A 1 × 3 SOI multimode interferometer (MMI), acting as beam splitter, is at the input of the system and its outputs are connected to the dielectric waveguides, thus enabling light propagation in each cavity and the biomarkers detection through the light collected by photodiodes at the output of the system. At the input/output there are dielectric waveguides to enhance the coupling efficiency between those waveguides and the single-mode fibers to be used to carry light from the optical source exciting the platform and to connect the outputs to the photodiodes. The beam splitter is also dielectric, mainly to minimize the insertion loss of that component.

The SOI technology has been selected for the dielectric components because of its low-loss, high-index contrast enabling ultracompact bent waveguides and MMI, the availability of high-efficiency fiber couplers in this technology, and especially due to its compatibility with the configuration of the plasmonic cavities. Due to these reasons, the biosensing platform in Figure 1 can be fully integrated on a SOI chip, using a CMOS-compatible fabrication process.

In the next sub-sections, the design and results of each component of the biosensing platform are reported.

### 2.1. Resonant Plasmonic Cavity

**Figure 2:**
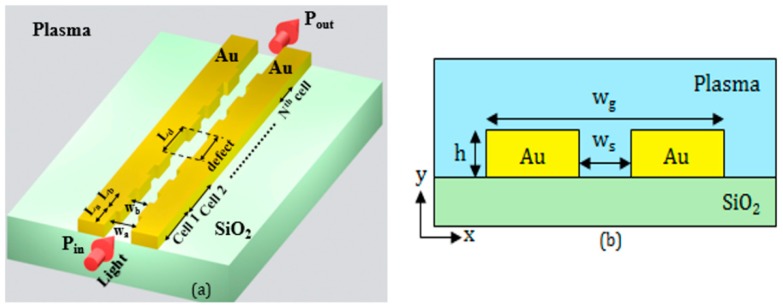
(**a**) Configuration of the plasmonic biosensor; (**b**) Cross-section of the plasmonic slotted waveguide.

The key element of the biosensing platform is the device sensing the target biomarkers. It is based on a plasmonic 1D Bragg grating, formed in an optimized MIM waveguide, and including a properly-engineered point defect [30], as shown in Figure 2a. Some preliminary numerical results on a similar plasmonic 1D Bragg grating for biosensing have been recently presented by the authors [31]. The results in [31] are based on two-dimensional simulations and are related to the single plasmonic cavity, while here we report on the design of the whole biosensing platform, and calculations are obtained by three-dimensional simulations.

The guiding structure (see the cross-section in Figure 2b) is formed by two closely-spaced gold (Au) rails, whose distance *w_s_* is periodically modulated. The rail height and the width of the whole guiding structure are denoted by *h* and *w_g_*, respectively. In our design, we assume that the gold rails are deposited on the buried oxide of the SOI substrate. The cover medium is the plasma where the biomarkers are dispersed.

Since the cavity is supposed to operate as an affinity label-free biosensor [3], gold has been chosen for the metal structure because it is an electrochemically inert material, is biocompatible, and is able to ensure a good stability of the chemical reaction between the antibody and the antigen [32], making the functionalization process easier.

Water has been assumed as the cladding material, because of the strong similarity of its optical properties with those of the plasma where the biological substances are dispersed.

In the selection of the operating wavelength, we have compared two possible values, 1550 nm and 1300 nm. At both wavelengths tunable laser diodes with low-cost, narrow-linewidth, stable, and sufficiently high output power are available. In addition, at both wavelengths the absorption loss of the silica substrate is negligible. The latter option (operating wavelength = 1300 nm) has been preferred because if we compare the water absorption at 1300 nm and 1550 nm we observe that it is significantly lower at 1300 nm.

The parametric analysis on both the MIM waveguide and grating was carried out by the 3D finite element method (FEM). The Lorentz-Drude model has been considered to define the wavelength-dependent gold dielectric constant [33], while the Palmer-Williams model [34] has been used to characterize the optical properties of the water. The dispersion of the SiO_2_ refractive index has been taken into account via the Sellmeier model [35].

The choice of the dielectric function for the metal has been an important design step. Although the cavity has not been fabricated, in our analysis we have considered a model for the optical properties of metal that best fit the experimental results. For this reason the Lorentz-Drude model has been chosen. It, unlike other models (e.g., Drude or Johnson and Christy), takes into account the interband transitions and, thus, the dielectric response of real metals [36], in order to make the numerical results more reliable and comparable with experimental data.

### 2.2. Design of the MIM Waveguide

The waveguide design was carried out aiming at maximizing both the confinement factor in the slot between the gold rails, which has a strong influence on the sensor sensitivity, and the propagation length, which is inversely proportional to the imaginary part of the complex propagation constant. The need of minimizing the sensor footprint was also taken into account.

The confinement factor *Γ* is the portion of the electromagnetic (e.m.) energy confined within the slot region and it is defined as:

(1) $$\Gamma =\frac{\iint_{slot}{\left|E(x,y)\right|}^{2}dxdy}{\iint_{\infty }{\left|E(x,y)\right|}^{2}dxdy}$$

where ***E*** is the electric field vector.

**Figure 3:**
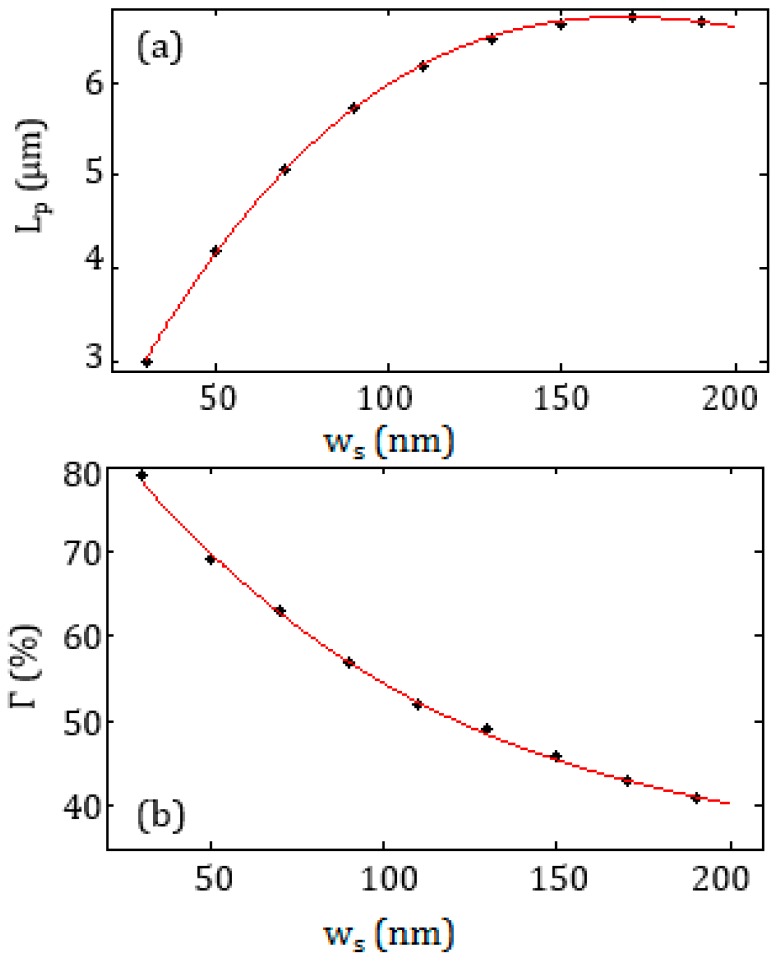
(**a**) Propagation length *L_p_* and (**b**) confinement factor *Γ* as a function of the waveguide width, *w_s_*.

The waveguide propagation length is the length where the intensity of propagating beam decreases to 1/e and it can be expressed as [36]:

(2) $$L_{p}=\frac{1}{2Im\left\{\beta \right\}}$$

where *β* is the complex propagation constant. In Figure 3a,b the behaviour of *L_p_* and *Г* as a function of *w_s_* are reported, assuming an initial value of *h* equal to 100 nm and *w_g_* = 450 nm (see Figure 2b). The value *w_g_* = 450 nm has been considered to minimize the device footprint. Smaller size was not considered because it could affect the slotted waveguide performance, due to some changes in the mode distribution, and the fabrication of the slot could become more challenging.

As shown from the results of FEM simulations reported in Figure 3, the confinement factor decreases as the slot width *w_s_* increases, because the plasmonic mode is less confined in the slot. This corresponds to an increase of the propagation length, due to lower optical losses. Thus, the maximization of the confinement factor demands low values of *w_s_* while the *L_p_* maximization requires large values of *w_s_*. As a trade-off between these conflicting needs, the slot widths *w_a_* = 150 nm and *w_b_* = 90 nm have been chosen to realize each cell of the Bragg grating. The two selected values of the slot width, i.e., *w_a_* = 150 nm and *w_b_* = 90 nm, allow a confinement factor *Г* > 40% and a propagation length *L_p_* > 5 μm.

**Figure 4:**
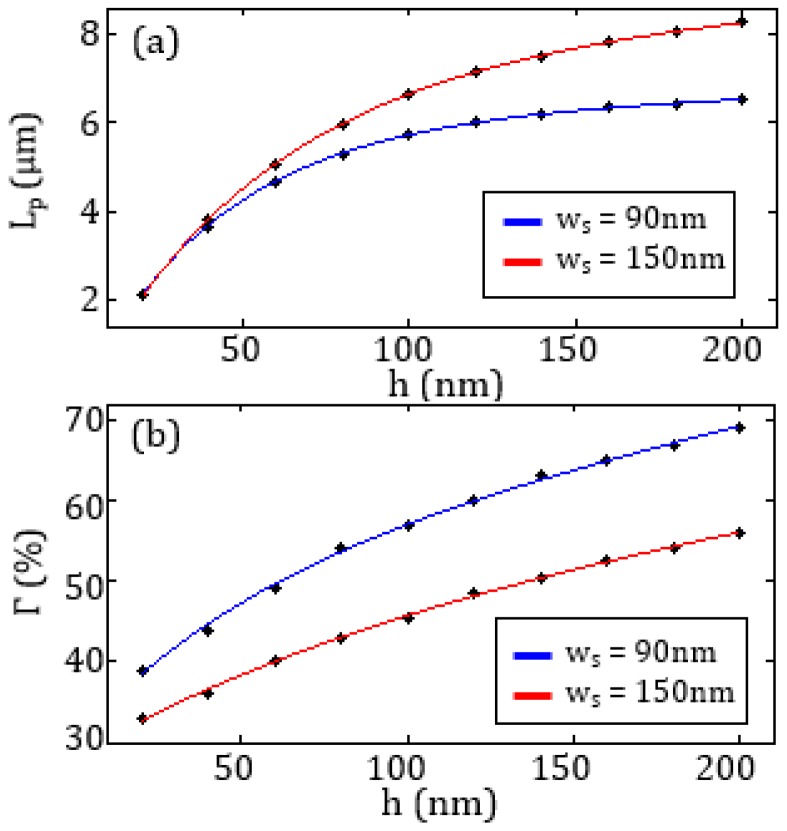
Behaviour of (**a**) *L_p_* and (**b**) *Г* as a function of *h* with *w_s_* = 90 nm (blue curve) and *w_s_* = 150 nm (red curve).

The dependence of *L_p_* and *Γ* on the waveguide height *h* has been also evaluated for two different values of *w_s_* (see Figure 4).

Large values of *h* provides higher values of the confinement factor and longer propagation length, but the fabrication of the slot is more challenging. Since the values of *L_p_* and *Г* are strongly affected by a thin Au layer, the value *h* = 100 nm has been assumed as the best compromise between the reduction of the etch depth and the need to maximize the performance of the plasmonic slotted waveguide.

**Figure 5:**
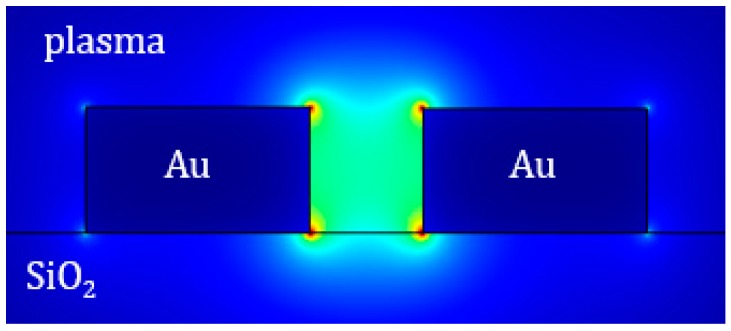
Electric field distribution for the quasi-TEM mode supported by the plasmonic slotted waveguide with *w_s_* = 90 nm and *h* = 100 nm.

As already reported in the literature [37,38], the optimized waveguide with *w_s_* = 90 nm or *w_s_* =150 nm and *h* = 100 nm, supports a quasi-TEM (Transverse Electromagnetic) mode well confined in the slot (*Γ* = 40% for *w_s_* = 90 nm). The field distribution relevant to that mode is shown in Figure 5. The good light confinement between two closely-spaced Au rails implies a strong interaction of the quasi-TEM propagating mode with the blood plasma where the analyte is dispersed.

### 2.3. Design of the Resonant Cavity

**Figure 6:**
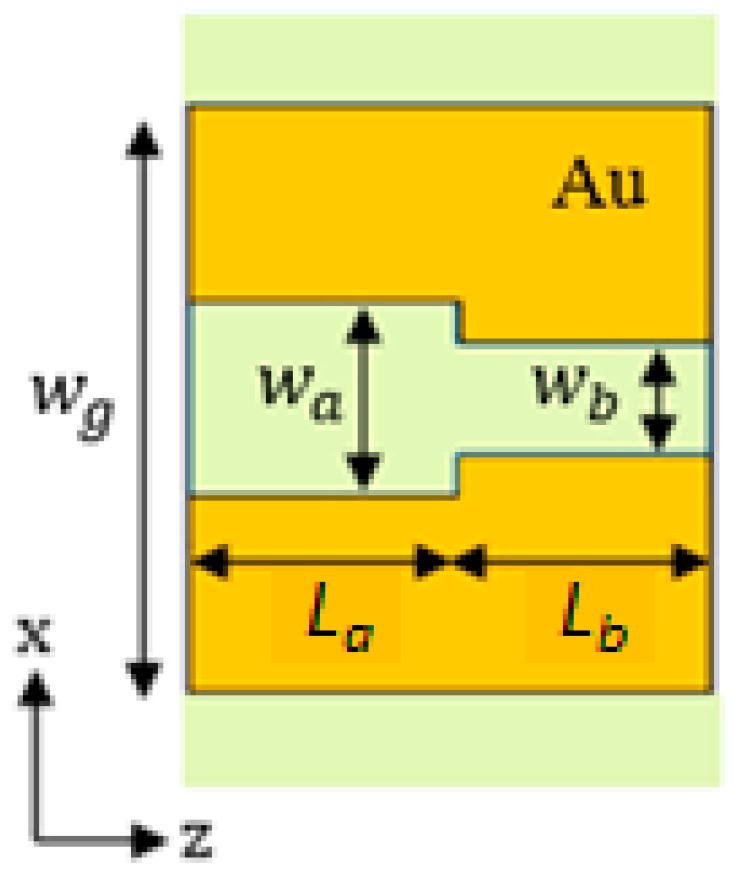
Top view of the Bragg grating unit cell.

The plasmonic cavity is based on a Bragg grating in the MIM waveguide that can be realized by simply modulating the slot width according to a square-wave profile. The unit cell of the grating, shown in Figure 6, is formed by two slot sections with different widths, *w_a_* = 150 nm and *w_b_* = 90 nm, and lengths, *L_a_* and *L_b_*.

The first-order grating was designed by using the Bragg condition [39]:

(3) LaRe{neff,a}+LbRe{neff,b}=λB/2

where *λ_B_* is the Bragg wavelength, and *n_eff,a_* and *n_eff,b_* are the effective indices of the quasi-TEM modes supported by the two sections of the grating unit cell. In particular, *n_eff,a_* is the effective index of the quasi-TEM mode supported by the section having the slot width equal to *w_a_* , while *n_eff,b_* is the effective index of the mode propagating in the section with the slot width *w_b_*. The lengths of the two sections of the unit cell, *L_a_* and *L_b_*, can be defined according to the following conditions that are derived from the Bragg condition [40]:

(4) La=λB4Re{neff,a}·Lb=λB4Re{neff,b}

The grating was designed in order to centre the photonic band gap at *λ_B_* = 1300 nm. Based on the results of the modal analysis, *w_a_* and *w_b_* were fixed to 150 nm and 90 nm, respectively, in order to achieve an index modulation able to guarantee the formation of the desired photonic band gap. The corresponding effective indices for the fundamental TEM modes are Re(*n_eff,a_*) = 1.6424 and Re(*n_eff,b_*) = 1.710, respectively. From Equation (4), the lengths of the two sections of the unit cell have been derived as *L_a_* = 198 nm and *L_b_* = 190 nm.

The number of the Bragg cells *N* has been selected in order to maximize the grating extinction ratio, i.e., the ratio between the maximum and the minimum in the grating transmission spectrum. High values of *N* correspond to a large band gap and a high extinction ratio, but at the expense of the transmission values (T < 10%) for the plasmonic cavity, which prevents the cavity from acting as a sensor.

**Figure 7:**
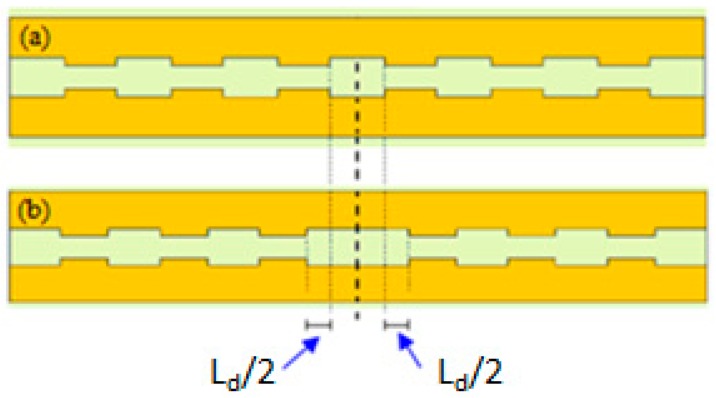
Top view of (**a**) the Bragg grating and (**b**) the plasmonic cavity with the defect to create the resonant behaviour.

The resonant cavity is formed by introducing a defect at the centre of the grating, as shown in Figure 7. The defect length is denoted as *L_d_*.

**Figure 8:**
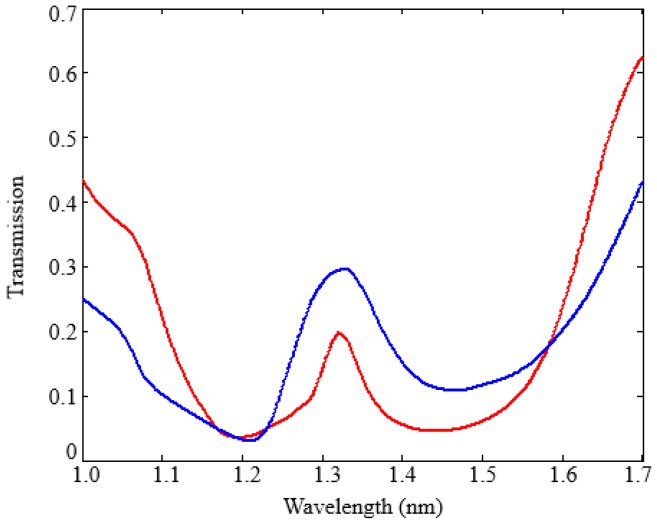
Transmission spectrum of the plasmonic cavity with *N* = 6 (blue curve) and *N* = 8 (red curve).

As shown in Figure 7, the cavity includes two identical reflectors, each one formed by *N*/2 unit cells with a defect in the centre of the device. The defect size and the number of the unit cell affects the resonance condition and the cavity performance, in terms of Q-factor and resonance transmission. We have assumed an optimized defect length *L_d_* = 190 nm to obtain a resonance condition around 1300 nm. Transmission spectra of the plasmonic cavity with different numbers of the unit cells *N* were calculated by 3D FEM simulations to define the best compromise in terms of the Q-factor and resonance transmission (see Figure 8). A Q-factor of about 20 with a resonance transmission T = 19% was obtained with *N* = 8, which is assumed as the best number of the unit cells. This result confirms a typical value of Q-factor obtained with plasmonic cavities (Q < 10^2^), together with the advantage of a very strong energy confinement and an appropriate value of the resonance transmission for an accurate measurement of the resonance condition. The detection resolution can be improved by decreasing the number of the unit cells, but at the expenses of a lower Q-factor. In particular a resonance transmission T = 32% has been calculated with *N* = 6, but the Q-factor is <10, which corresponds to a clear worsening of the sensor sensitivity.

We have verified that an increase of *w_a_* and *w_b_* induces a decrease of the Q-factor and an increase of the resonance transmission, while a worsening of T has been calculated by decreasing both *w_a_* and *w_b_* with an improvement of Q. This last condition makes the device fabrication too difficult. Therefore, *w_a_* = 150 nm, *w_b_* = 90 nm, *L_a_* = 198 nm, *L_b_* = 190 nm, *L_d_* = 190 nm, and *N* = 8 have been assumed as the optimal cavity parameters to obtain the best compromise between *T* and Q (Q ~ 20 and *T* = 19%). This value of resonance transmission corresponds to an optical loss of each plasmonic biosensor of *α_cavity_* = 7.2 dB. We have verified that, if the operating wavelength is changed from 1330 nm to 1550 nm, the nanocavity performance in terms of Q-factor and T worsens because the water absorption increases, and *L_a_* and *L_b_* increase (see Equation (4)) and, consequently, the optical loss increases.

We have also analysed the effect of fabrication tolerance on *L_a_* and *L_b_* on the nanocavity performance. The analysis is based on accurate 3D FEM simulations. The results of this activity, which are summarized in Table 1, confirm that slight changes of *L_a_* and *L_b_* have no remarkable effects on the nanocavity performance in terms of Q-factor and T.

### 2.4. Design of the MMI

The optical MMI has been used to convey the light in several optical paths. In particular, the design of the specific device we are considering includes three different plasmonic cavities and, therefore, a 1 × 3 MMI. A dielectric configuration of the MMI has been chosen, instead of a plasmonic one, in order to reduce the optical losses and to simplify the mode conversion in the plasmonic slot configuration. In fact, several plasmonic MMIs with a very compact footprint have been proposed in the literature [41], but they are usually affected by high optical losses and more complex fabrication processes. On the contrary, dielectric MMIs have demonstrated low optical losses together with high reliability, although with larger device footprints [42,43].

SOI technology has been assumed for the MMI, due to high fabrication yield and low optical losses. The 1 × 3 MMI has a configuration similar to that proposed in [43], which shows only two optical outputs, but with a very compact footprint. We have designed the MMI to obtain a symmetric light splitting in three different branches at the output, thus changing both the length and the width of the optical device.

**Figure 9:**
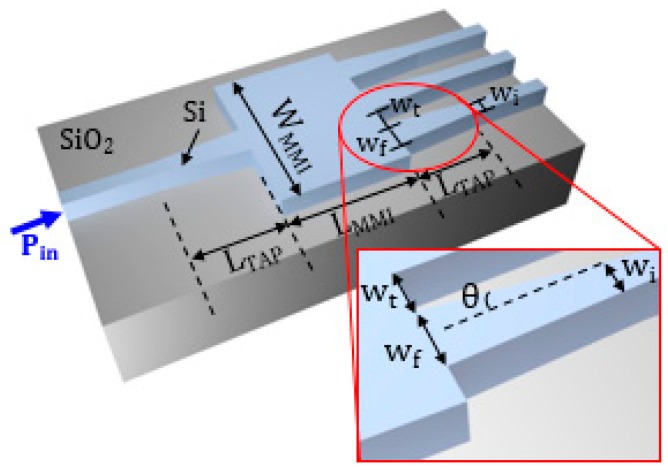
Configuration of the 1 × 3 SOI MMI with a focus on the tapering section in the inset.

The 3D beam propagation method (BPM) has been used to define the optimal configuration of the 1 × 3 SOI MMI (see Figure 9), corresponding to the minimum optical loss and the most compact footprint. Despite the footprint of MMI is >200 μm^2^, this numerical method allows for obtaining accurate results with restrained time consumption.

The highest performance has been obtained with a total width *W_MMI_* = 5.4 μm and a length *L_MMI_* = 37 μm. A tapering section has been introduced at the input and each output of the MMI to improve the light coupling. The input taper is specular to that at the output and it has an initial waveguide width of 500 nm and a thickness of 220 nm. The width of the waveguide linearly increases up to 1.2 μm with a taper length of 5 μm, which corresponds to a taper angle θ = 6.8°. The distance between each output branch is equal to 1.3 μm, in order to avoid any coupling. A PMMA cladding (*n* = 1.48 at *λ* = 1320 nm) has been considered to minimize the optical losses, particularly in the mode converter (see Section 2.5) placed between the MMI and the plasmonic cavity.

**Figure 10:**
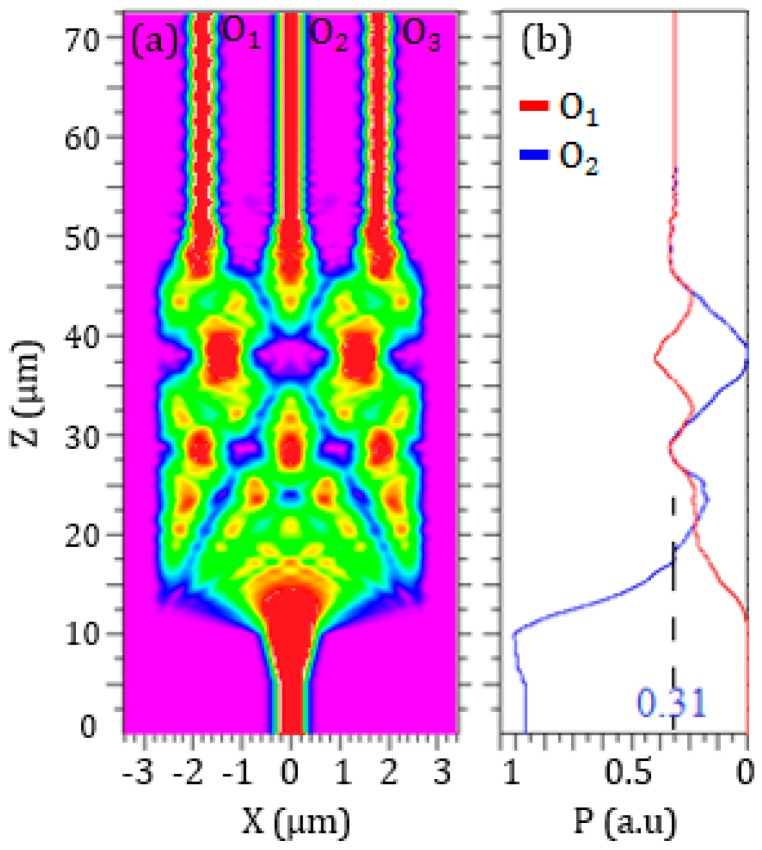
3D BPM simulations of the 1 × 3 MMI in SOI technology. (**a**) Light propagation and (**b**) power distribution in the MMI and at the output waveguides. O_1_ is the power in the optical path from the input to a lateral output branch, O_2_ is the optical path related to the central output branch.

A total MMI optical loss *α_MMI_* = 0.28 dB has been calculated. This value corresponds to an optical power at the output of each MMI branch equal to 31% of the optical power at the MMI input, as shown in Figure 10.

This MMI configuration has a total footprint of about 250 μm^2^.

### 2.5. Design of the Mode Converter

**Figure 11:**
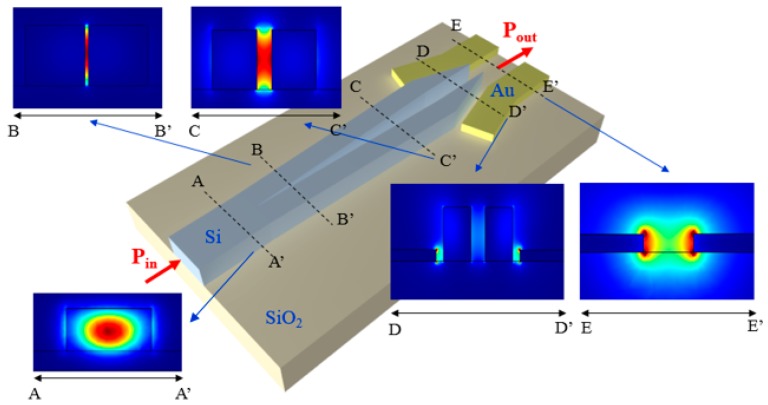
Configuration of the hybrid mode converter proposed in [44], with 3D FEM simulations with the energy confinement in the insets calculated in different regions of the mode converter.

A mode converter between the MMI and the plasmonic cavity is necessary to gradually convert the mode propagating in the dielectric optical waveguide into the mode of the plasmonic slotted structure, in order to minimize the optical losses. Another mode converter is included in each channel to convert the plasmonic mode into the guided mode in the waveguide (see Figure 1). We have considered the configuration of the mode converter proposed in [44]. The structure includes a tapered slot in the silicon waveguide, which directly ends in the plasmonic slot, as shown in Figure 11.

At the input of the converter, we have assumed a silicon waveguide with a cross-section of 500 nm × 220 nm. This is the same size of the MMI output waveguides. The Au slotted structure is 100 nm thick and 150 nm large. The total length of the mode converter is 10 μm. 3D FEM simulations have been carried out to calculate the performance of the mode converter at *λ* ~ 1320 nm, corresponding to the resonance condition of the plasmonic cavity. A strong energy confinement has been observed in the silicon tapered slot, thus improving the coupling between the dielectric waveguide and the plasmonic cavity.

Assuming a power of 1 mW at the input of the mode converter, we have calculated an optical power of 0.51 mW at the output of the device, which corresponds to an optical loss of the mode converter *α_MC_* of about 3 dB. Another mode converter with the same configuration has been placed at the output of the plasmonic cavity to allow the light propagation in a larger dielectric waveguide, thus minimizing again the optical losses.

The minimum distance between the plasmonic cavities in the direction orthogonal to the propagation direction has been assumed 50 μm, in order to allow the functionalization of each cavity with different biomarkers. This also affects the device footprint because longer waveguides between the MMI outputs and the inputs of the mode converters are required in order to minimize the bending losses relevant to the bent waveguides connecting the MMI and the plasmonic cavities. All the geometrical features of the sensing platform are summarized in Table 2.

Therefore, the whole pattern of the sensing platform (see Figure 1), which includes the 1 × 3 SOI MMI and the mode converters at the input and output of the plasmonic cavities, has a total footprint of 116.3 μm × 100 μm = 0.011 mm^2^, confirming the small size of the biosensing platform.

The total on-chip optical losses *α_TOT_* related to each biosensor are given by:

(5) $$\alpha_{TOT}=\alpha_{MMI}+\;\alpha_{MC}+\alpha_{cavity}+\alpha_{MC}\;=18\;\mathrm{dB}$$

This result confirms that a signal can be clearly detected at the output, even if the optical losses are remarkable due to the presence of the metal structure, thus confirming the advantage of the proposed cavity for the biosensing application. For instance, with *P_in_* = 10 mW (a value that can be achieved by using commercial lasers), at resonance, we can obtain an optical power of about 1.6 mW and 0.3 mW at the input and at the output of each plasmonic cavity, respectively. Due to the loss of the mode converter, at resonance, the optical power at the output of each output waveguide (*P_out-_*_1_, *P_out-_*_2_, *P_out-_*_3_) is approximately 0.15 mW. That value can be easily measured by a photodiode. In the plasmonic cavity the 40% of the input power is confined in the slot, where the vast majority of the light/matter interaction takes place.

## 3. Design of the Label-Free Biosensor

As already mentioned, the platform has been designed for label-free biosensing of several protein biomarkers. The operation principle of the surface sensing is based on changes of the average thickness *t* of the functionalization layer, when the biomarker molecules bind to the bioreceptor molecules, so providing changes in optical properties (i.e., resonance shift and resonance transmission changes) [45]. The initial thickness of the functionalization monolayer (*t*_0_) used for the binding with the target protein has been assumed equal to 10 nm, which is a typical value measured experimentally [46]. The average thickness *t* of the functionalization layer is *t*_0_ + *Δt*, being *Δt* the average thickness increase to the bioreceptors-target molecules binding.

After the interaction with the target biomarkers, the adlayer becomes irregular, with an increase of thickness of a few nm where the bioreceptors-target molecules binding occurs.

**Figure 12:**
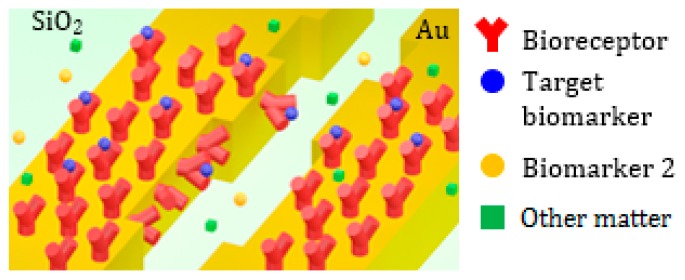
Schematic of the surface biosensing in the plasmonic cavity for selective detection of a target biomarker.

The average thickness *t* of the adlayer depends on the concentration of the target biomarker in the biologic fluid interacting with the nanocavity. The surface sensing is sketched in Figure 12, where the selective binding between the target proteins and the bioreceptor molecules attached to the cavity surface is depicted.

**Figure 13:**
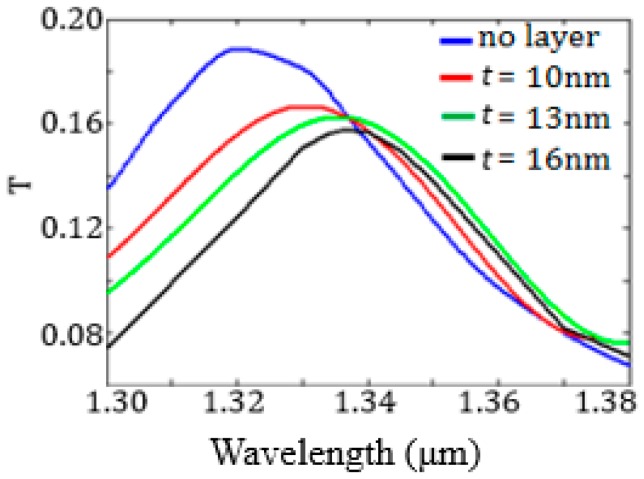
Transmission spectra of the plasmonic biosensor as a function of thickness *t*, corresponding to different values of biomarker concentration.

The cavity spectral response has been evaluated by 3D FEM simulations for *t* ranging from 10 nm to 16 nm (see Figure 13).

As expected, the resonance wavelength increases as *t* increases, while the resonance transmission decreases. The sensor surface sensitivity *S_S_*, equal to the derivative of the resonance wavelength with respect to *t*, has been numerically evaluated by monitoring the shift of the resonance wavelength due to the *t* increase and corresponds to *S_S_* = 1.8 nm/nm.

The detection limit (DL) of the biosensor is equal to [24]:

(6) $$DL=\frac{n_{fl}-n_{c}}{\partial n_{fl}/\partial c}\frac{\Delta \lambda_{\min}}{S_{S}}$$

where *n_fl_* is the refractive index of the functionalization layer (=1.45, as in [5,6]), *n_c_* is the refractive index of the cover medium (blood plasma), *c* is the biomarker concentration in that solution, and *Δλ_min_* is the minimum detectable shift in the resonance wavelength. It has been demonstrated by ellipsometric experimental studies that a good approximation of ∂*n_fl_*/∂*c* for most of proteins is 187 mm^3^/g [47,48]. As in [49], we have assumed that the optical setup used for measurements can detect *Δλ_min_* of FWHM/200, where FWHM (=*λ_res_*/Q) is the full-width at half-maximum of the nanocavity. The calculated DL value is 128 pg/mm^2^. Although this value is worse than that obtained in integrated optical biosensors at the micro-scale, the device we propose has a footprint much smaller than those and satisfies the requirements of several applications, e.g., detection/monitoring of coronary artery disease though the detection of C-reactive protein, β_2_-microglobulin, and adiponectin, as discussed at the end of this section, and also provides a very compact footprint.

The biosensor resolution can be estimated as the ratio between the minimum biomarker mass *m* that can be detected by the device and the volume *V* of the plasma directly interacting with the functionalization monolayer. The minimum mass can be written as *m* = DL × *A*, being *A* the sensor footprint (=1.57 μm^2^), and it is equal to 0.2 fg. The volume *V* is given by the product between *A* and the thickness *t_S_* of the fluid interacting with the sensor. Assuming *t_S_* = 300 μm [49], we have calculated a sensor resolution of 0.42 μg/mL.

The dynamic range of the biosensor is another important performance parameter. In fact, a large range in which the resonance shift changes linearly with the values of the biomarker concentration is necessary for accurate measurements, so allowing the monitoring of disease progress or the efficiency of the drug treatments.

**Figure 14:**
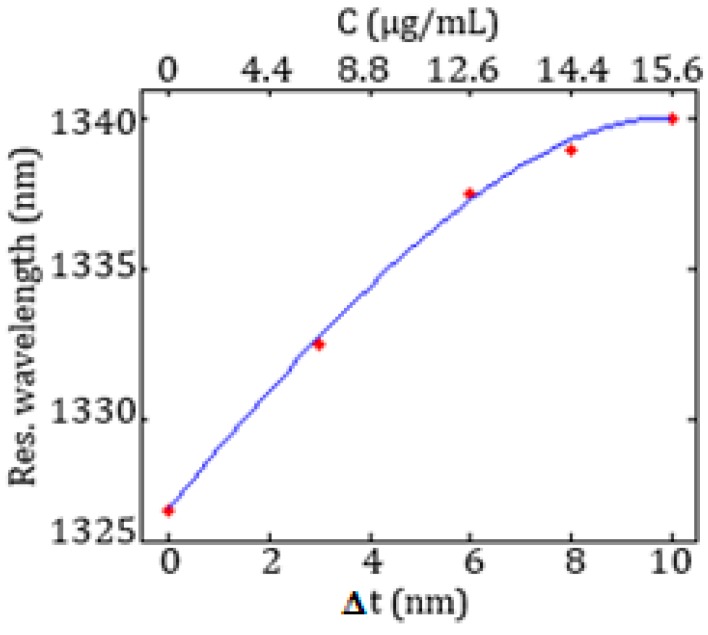
Resonance wavelength as a function of *Δt* and concentration.

In Figure 14 the resonance wavelength as a function of the biomarker concentration *C* and of the increase *Δt* of the average thickness of the functionalization layer due to the bioreceptors-target molecules binding is shown.

A linear behaviour between the resonance shift and the biomarker concentration can be observed up to 12.6 μg/mL.

We have compared the performance of the sensor proposed in this paper, in terms of DL and footprint, to the state-of-the-art, in order to demonstrate its main advantage.

In fact, label-free integrated optical biosensors based on interferometers, especially Mach-Zehnder and Young devices, have a very good DL (down to 0.01 pg/mm^2^), but with a footprint of a few mm^2^. Ring resonator photonic biosensors have a footprint of the order of 10^3^ μm^2^ and a DL down to 0.06 pg/mm^2^ [5,49]. Photonic crystal (PhC) biosensors exhibit a footprint of approximately 10–20 μm^2^ but their DL is of several tens of pg/mm^2^. Finally, plasmonic biosensors [17,24] have very compact footprint, but they are less efficient than microphotonic biosensors.

In Table 3, the performance of the designed sensing element (plasmonic nanocavity) is compared to that exhibited by some resonant photonic and plasmonic biosensors at the state-of-the-art in different technologies.

The Table 2 shows that the main advantage of the designed biosensor is relevant to its extremely high compactness (footprint of approximately 1.6 μm^2^), with a DL value appropriate for several applications. This confirms the suitability of the proposed cavity as efficient biosensor for multiplexed analysis of several biomarkers, due to its ease of integration in lab-on-chip systems.

### An Example of Potential Application: Detection and Monitoring of Coronary Artery Diseases

In this subsection, we report on an important biomedical application of our sensing platform. We refer to the early detection/monitoring of the coronary artery disease, which causes about 40% of deaths due to cardiovascular disorders, the first cause of death all over the world [52]. Biomarkers indeed allow clear clinical picture of the disease stage and useful information about its history and predictive progress [53]. However, most of biomarkers, including those for artery disease detection, are non-specific and their altered levels may be associated to several pathologies. Therefore, a simultaneous multi-analysis of different biomarkers represents the best approach to have a complete clinical picture of a specific disease, in order to define its progress and the drug efficiency [54].

As already mentioned the selected biomarker panel for early detection/monitoring of the coronary artery disease includes β_2_-microglobulin, C-reactive protein, and adiponectin. Since the reference value of these biomarkers in healthy patients are 0.5 μg/mL [55], <1 μg/mL [56], and >4.39 μg/mL (for men), and >6.84 μg/mL (for women) [57], respectively, we can state that our biosensor resolution, 0.42 μg/mL, is well suitable to detect abnormal values of those biomarkers.

Since the surface functionalization of each plasmonic cavity is necessary to allow the specific detection of these biomarkers. Anti-human C-reactive protein antibody [58] and anti-human β_2_-microglobulin [59] can be used as functionalization layers for C-reactive protein and β_2_-microglobulin, respectively, and the FC-gamma receptor [60] for the detection of adiponectin, due to the highest value of binding affinity of those proteins, corresponding to a higher sensitivity and a larger dynamic range of the biosensor.

## 4. Conclusions

The design of a new ultracompact resonant plasmonic multi-analyte label-free biosensing platform for high-resolution detection of protein biomarkers has been proposed. The platform includes a number of biosensors, each of them based on a plasmonic nanocavity, consisting of a periodic metal structure to be deposited on a silicon oxide substrate. The resonant component is a plasmonic Bragg grating in a MIM waveguide including a 190 nm-long defect. A very small footprint 1.57 μm^2^ and a strong light-matter interaction enabling a resolution of 0.42 μg/mL, which is compliant with a number of medical applications, has been achieved. The design of an ultracompact (footprint ~ 0.011 mm^2^) biosensing platform for a multiplexed analysis with a simultaneous detection of three biomarkers for detecting and monitoring coronary artery diseases has also been proposed. The biosensor performance confirms the suitability of the proposed platform for several applications in biomedical environments, and not only limited to the detection of cardiovascular diseases.

The proposed device could be used for point-of-care diagnostics due to its ease of integration in lab-on-chip systems. Moreover, the use of optical signals for the biomarkers detection, together with the achieved values of sensitivity would enable carrying out medically-accurate and fast tests from a single blood drop. Another advantage of the designed sensing platform is related to the simultaneous detection of several biomarkers, which improves the efficiency of the medical analysis, reducing false-positive cases, which can be obtained more frequently than with a single biomarker detection. This improves the prevention and reduces the waiting time for the beginning of the therapy for each patient, also minimizing the hospital stay of false-positive patients, who do not require any treatment.

## Figures and Tables

**Table 1:** Effect of fabrication tolerance on *L_a_* and *L_b_* on the nanocavity performance.

*L_a_*	*L_b_*	Q-Factor	T
198	190	20	19%
200	190	18	17%
190	190	15	14%
200	200	16	16%

**Table 2:** Geometrical features of the biosensing multiplatform.

Geometric Feature	Size (μm)
Length of the input waveguide	3
Length of the MMI	37
Length of the waveguides from the MMI outputs to the mode converter inputs	50
Length of the mode converters	10
Length of the plasmonic nanocavities	3.3
Length of the output waveguides (from the output of the mode converters to the outputs of the platform)	3
Distance between the nanocavities in the direction orthogonal to the propagation one	50

**Table 3:** State-of-the-art of integrated photonic/plasmonic biosensors.

Publication Year	Device Configuration/Technology	A (μm^2^)	DL (pg/mm^2^)
2010	SOI ring resonator [49]	1600	0.3
2011	Nanolaser in a slab 2D PhC [50]	10	21
2011	Slotted photonic crystal cavity [51]	>20	60
2013	Circular plasmonic interferometer array [22]	22500	0.4
2015	Si_3_N_4_ ring resonator [5]	2200	0.06
2015	Plasmonic Bragg grating [24]	660	7.9
2016	Plasmonic Bragg grating with defect (this paper)	1.57	128
